# Supplementary material for: Toxicity and Control Efficacy of an Organosilicone to the Two-Spotted Spider Mite Tetranychus urticae and Its Crop Hosts
Source: Insects. 2022 Mar 30;13(4):341. doi: 10.3390/insects13040341 (PMC9028738; doi:10.3390/insects13040341)
Supplement: Supplementary file 1 [file insects-13-00341-s001.zip › insects-1636776-supplementary.pdf]

Table S1. Phytotoxicity index of strawberry, eggplant, kidney bean, and cucumber caused by the spray of Silwet 408.

Con. (mg/L)	Tem. (°C)	Phytotoxicity Index											
		Strawberry			Eggplant			Kidney Bean			Cucumber		
		Day 1	Day 3	Day 7	Day 1	Day 3	Day 7	Day 1	Day 3	Day 7	Day 1	Day 3	Day 7
4000	35	66.1 ± 8.5	39.7 ± 6.8	18.2 ± 6.0	64.3 ± 6.6	75.3 ± 7.1	72.7 ± 3.9	72.2 ± 9.4	92.6 ± 6.0	86.3 ± 2.6	67.9 ± 2.7	84.0 ± 4.0	82.9 ± 5.2
	30	35.8 ± 4.6	24.2 ± 4.1	6.9 ± 0.6	72.6 ± 2.6	83.5 ± 4.1	77.8 ± 0.0	54.3 ± 9.6	64.2 ± 8.3	88.3 ± 2.2	74.8 ± 4.2	82.0 ± 2.1	82.6 ± 2.1
	25	58.5 ± 10.9	33.7 ± 2.1	14.5 ± 3.5	69.5 ± 2.6	81.0 ± 5.8	71.2 ± 3.4	53.7 ± 12.1	64.8 ± 8.0	77.8 ± 0.0	69.1 ± 4.4	79.3 ± 3.7	78.8 ± 2.8
	20	41.3 ± 5.3	31.3 ±6.0	15.2 ± 2.9	71.1 ± 6.0	75.3 ± 6.2	70.5 ± 5.4	46.3 ± 7.6	64.8 ± 9.2	87.0 ± 4.0	69.1 ± 3.6	82.7 ± 2.0	83.3 ± 3.3
2000	35	19.7 ± 10.1	21.7 ± 11.6	8.0 ± 1.3	27.8 ± 2.6	36.9 ± 11.2	35.4 ± 10.3	21.6 ± 4.3	20.4 ± 3.3	38.0 ± 5.3	44.4 ± 8.7	30.9 ± 8.5	30.9 ± 8.5
	30	18.7 ± 2.9	13.0 ± 4.0	4.8 ± 1.7	31.8 ± 4.1	37.1 ± 5.9	44.2 ± 1.4	22.5 ± 13.5	22.5 ± 13.5	46.3 ± 2.3	17.9 ± 1.8	16.7 ± 4.0	16.7 ± 4.0
	25	11.6 ± 1.7	6.2 ± 1.0	2.9 ± 0.2	25.8 ± 3.9	40.7 ± 6.1	47.1 ± 3.5	19.4 ± 6.0	20.4 ± 3.8	41.7 ± 3.5	23.5 ± 2.8	33.2 ± 4.9	33.8 ± 4.3
	20	33.1 ± 12.0	21.4 ± 8.9	10.4 ± 1.9	29.9 ± 8.5	30.2 ± 5.1	35.4 ± 3.7	7.4 ± 0.8	19.4 ± 1.3	38.0 ± 5.3	28.3 ± 6.9	37.5 ± 5.0	37.7 ± 4.8
1000	35	10.1 ± 7.3	5.0 ± 2.8	2.5 ± 1.8	17.6 ± 6.5	14.6 ± 5.5	21.1 ± 5.2	2.8 ± 1.3	4.6 ± 1.5	14.8 ± 3.0	3.1 ± 1.0	3.7 ± 0.9	3.7 ± 0.9
	30	4.8 ± 1.8	4.4 ± 2.2	1.2 ± 0.5	14.2 ± 3.3	10.5 ± 1.0	15.1 ± 5.5	7.4 ± 1.5	5.6 ± 0.0	17.6 ± 2.0	1.2 ± 0.5	2.5 ± 1.3	3.1 ± 1.0
	25	6.0 ± 0.4	3.0 ± 0.3	2.8 ± 0.4	17.7 ± 5.5	12.1 ± 2.3	22.0 ± 3.0	3.7 ± 1.5	3.7 ± 0.8	10.7 ± 3.3	3.1 ± 1.3	3.7 ± 1.7	4.3 ± 1.3
	20	4.9 ± 1.3	1.3 ± 0.4	2.3 ± 1.2	12.1 ± 2.2	10.5 ± 4.4	15.0 ± 1.7	9.3 ± 4.0	19.4 ± 5.7	26.5 ± 4.1	0.6 ± 0.5	4.4 ± 0.9	3.2 ± 1.0

Con., concentration; Tem., temperature.

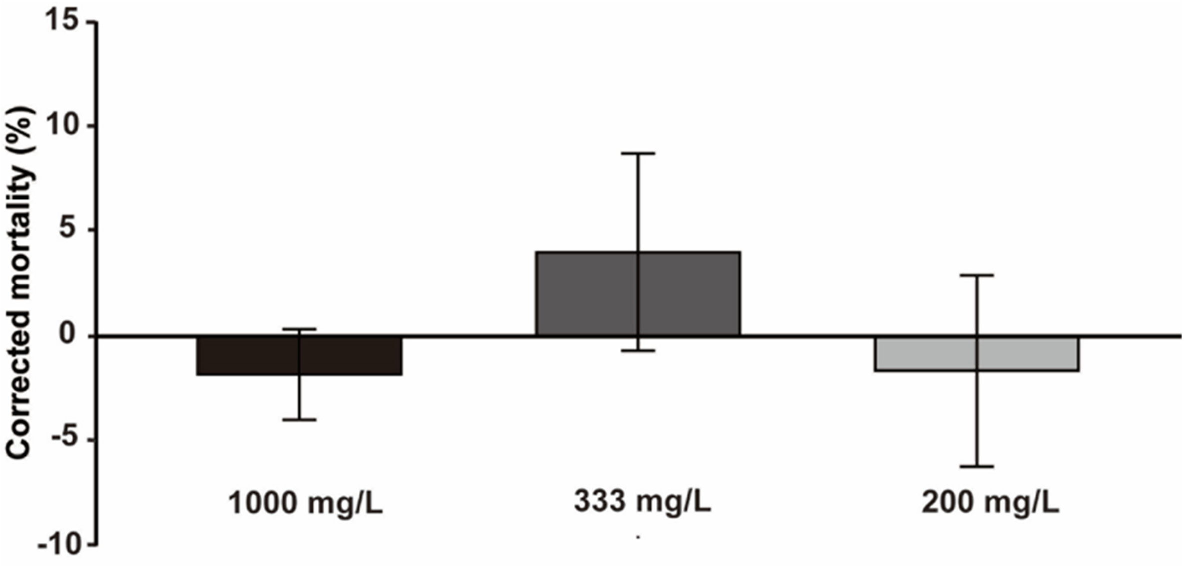

Figure S1 Corrected mortality of *Tetranychus urticae* eggs treated with three concentrations of Silwet 408 aqueous solution. For the corrected mortality measure, see text.
